# Effect of Diabetes on Post-stroke Recovery: A Systematic Narrative Review

**DOI:** 10.3389/fneur.2021.747878

**Published:** 2021-12-14

**Authors:** Seoyon Yang, Mathieu Boudier-Revéret, SuYeon Kwon, Min Yong Lee, Min Cheol Chang

**Affiliations:** ^1^Department of Rehabilitation Medicine, Ewha Womans University Seoul Hospital, Ewha Womans University School of Medicine, Seoul, South Korea; ^2^Department of Physical Medicine and Rehabilitation, Centre Hospitalier de l'Université de Montréal, Montreal, QC, Canada; ^3^Department of Dermatology, Ewha Womans University Seoul Hospital, Ewha Womans University School of Medicine, Seoul, South Korea; ^4^Department of Rehabilitation Medicine, College of Medicine, Yeungnam University, Gyeongsan, South Korea

**Keywords:** diabetes, stroke, recovery, function, outcome

## Abstract

**Background:** Patients with stroke often have comorbid diabetes. Considering its detrimental effects on brain function, diabetes may increase the risk of poor recovery.

**Methods:** The aim of this review was to investigate the effect of diabetes on post-stroke recovery by a systematic review. Several specific aspects of post-stroke recovery, including activities of daily living (ADL), motor, cognitive, and quality of life (QOL) recovery, were examined. We searched the PubMed, SCOPUS, Embase, and Cochrane Library databases for relevant studies on the effect of diabetes on post-stroke recovery, published until May 26, 2021. A total of 52,051 potentially relevant articles were identified. After reading the titles and abstracts and assessing their eligibility based on full-text articles, 34 publications were included in this review.

**Results:** Of 29 studies that assessed ADL recovery after stroke, 22 studies suggested that diabetes had a negative effect on recovery of ADL after stroke. Regarding motor recovery, only one out of four studies showed that diabetes had some effect on motor recovery after stroke. Of the two studies on cognitive recovery, one reported that diabetes was an independent predictor of poor cognitive recovery after stroke. Three studies on QOL reported that a poor QOL after stroke was associated with the presence of diabetes.

**Conclusions:** The current review suggests that the post-stroke recovery of ADL seems to be poorer in patients with diabetes than patients without diabetes. Further, there are insufficient data to conclude the effect of diabetes on motor and cognitive recovery, but it may have some influence on the quality of life after stroke.

**Systematic Review Registration:** doi: 10.37766/inplasy2021.11.0032, identifier: INPLASY2021110032.

## Introduction

Stroke is the second leading cause of death worldwide (1), and it is the main cause of major disability (2). While the incidence of stroke-related death is decreasing, the number of stroke patients is constantly increasing globally, in relation to aging and continued population growth (3). Most patients who experience stroke regain a certain degree of motor and functional capability. However, some patients may suffer from continuous deterioration in disability, and approximately one-third of all stroke patients remain dependent on supportive care (4). The disabilities caused by stroke can be devastating and often result in significant reduction in quality of life (QOL). The disabilities include weakness of limbs, postural imbalance, gait, loss of dexterity, and other various conditions associated with functional limitations (1).

Among multiple risk factors for stroke development, diabetes mellitus (DM) is a major risk factor for stroke, and approximately 20%−33% of patients with acute stroke have comorbid diabetes (5). Diabetes is a chronic metabolic disease associated with increased morbidity and mortality. In Type 1 DM (T1DM), the pancreas is not able to produce sufficient insulin due to the loss of beta cells, whereas in Type 2 DM (T2DM), the body is resistant to insulin, and the cells fail to respond to insulin properly (6). Diabetes is associated with many cardiovascular risk factors, such as hypertension, hyperlipidemia, obesity, and insulin resistance (7). It causes atherosclerotic changes in blood vessels at various locations, triggering macrovascular complications (stroke and coronary vascular or peripheral artery disease) and microvascular complications (diabetic neuropathy, nephropathy, or retinopathy) (8).

In patients with diabetes, inefficient glucose metabolism may cause negative impact on brain metabolism and function. Considering the detrimental effects of diabetes on brain function, it is postulated that diabetes impairs cortical plasticity and neural recovery after stroke (9). Stroke patients often suffer from residual impairment of function and difficulties in performing activities of daily living (ADL). ADL include the basic tasks that a person performs to function on daily basis, which include bathing, dressing, eating, grooming, toileting, and transferring (10). ADL limitations are defined when a person needs assistance with at least one task and when a person shows to inability to complete any ADL alone (11). Among the common causes of ADL limitations, which include older age, fractures, and heart disease, diabetes also causes ADL limitations (12), which may result in poor overall recovery after stroke.

Previous studies have evaluated the association between diabetes and post-stroke recovery; some of these have shown that diabetes is associated with poor recovery (13–17) while others have reported that no significant differences in recovery were observed in stroke patients with or without diabetes (7, 18–21). To date, the effect of diabetes on post-stroke recovery remains unclear. Thus, the aim of this review was to investigate the effects of diabetes on post-stroke recovery.

## Materials and Methods

This review follows the Preferred Reporting Items for Systematic Reviews and Met-Analyses (PRISMA) statement (22). The protocol of this meta-analysis was registered on INPLASY (International Platform of Registered Systematic Review and Meta-analysis Protocols) with a registration number of INPLASY2021110032. Two examiners (SY, MC) managed all aspects of title selection, data extraction, and analyses, independently. Any disagreements were resolved through discussion. We searched the PubMed, SCOPUS, Embase, and Cochrane Library databases for relevant studies published until May 26, 2021. To identify potentially relevant articles, combinations of the following key search phrases were used: “stroke,” “diabetes,” “outcomes,” “recovery,” “cognition,” “cognitive impairment,” “memory,” “motor,” and “recovery outcomes.” The following inclusion criteria were applied for the selection of articles: (1) enrollment of patients with acute stroke, including ischemic or hemorrhagic strokes, (2) patients diagnosed with either T1DM or T2DM, and (3) examination of the impact of diabetes on recovery, including specific domains, such as ADL, motor improvement, cognitive improvement, and QOL. Subtypes of stroke included both ischemic and hemorrhagic stroke. We excluded studies on chronic stroke, studies that involved adolescents or children, studies that did not include patients with diabetes, and studies that did not focus on the recovery of patients after stroke. We only included studies that specifically mentioned the impact of diabetes on the recovery of ADL, motor function, or cognition. Additionally, this review was limited to human studies, i.e., animal studies were not included; moreover, review articles, commentaries, letters, and case reports that did not present original data were also excluded. The methodological quality of the included studies was assessed using the Newcastle-Ottawa scale (NOS), which comprises the following three aspects: selection of subjects, comparability of groups, and assessment of outcome. The quality of each study was graded as low (0–3), moderate (4–6), or high (7–9) (23).

In the studies included, recovery of ADL after stroke was assessed using the following assessment tools: modified Rankin scale (mRS), functional independence measure (FIM), and modified Barthel index (MBI). The mRS was used to assess the functional status of stroke patients (18). It is a 6-item scale that assesses the degree of disability or dependency in ADL (24). In many studies, an mRS score higher than 2 or 3 was defined as poor ADL recovery (0: no symptoms; 1: no significant disability, able to carry out all usual activities despite some symptoms; 2: slight disability, able to look after one's own affairs without assistance but unable to carry out all previous activities; 3: moderate disability, requiring some help but able to walk unassisted; 4: moderately severe disability, unable to attend to one's own bodily needs without assistance and unable to walk unassisted; 5: severe disability, requiring constant nursing care and attention, bedridden, and incontinent; 6: dead) (25). The FIM was used to evaluate how disabilities affect ADL or a given activity. The FIM assesses the degree of disability depending on the patient's score in 18 items, including self-care, mobility, locomotion, communication, and cognition. The scores are rated on a 7-point scale, with the final score ranging from 18 (total dependency) to 126 (independency) (24). The MBI was used to determine whether patients can perform basic ADL, including functional mobility. MBI scores range from 0 to 100 points and represent the amount of severity: 0–40 (severe), 40–60 (moderate), and 60–100 (mild functional impairment) (26).

Other aspects of post-stroke recovery were assessed using the following tools. Motor recovery was assessed using the Fugl–Meyer assessment (FMA) scale, motricity index (MI), modified Brunnstrom classification (MBC), and functional ambulation category (FAC). Cognitive recovery was assessed using the mini-mental state examination (MMSE), which includes tests of orientation, memory, language, and attention. MMSE scores range from 0 to 30 points. Cognitive disability is defined according to educational level (junior high school and above: ≤ 24 points) (26). The health-related quality of life (QOL) was evaluated using the Medical Outcomes Study 36-Item short-form (SF-36) health survey and stroke-specific QOL scores.

## Results

### Study Selection and Risk of Bias

**Figure 1:**
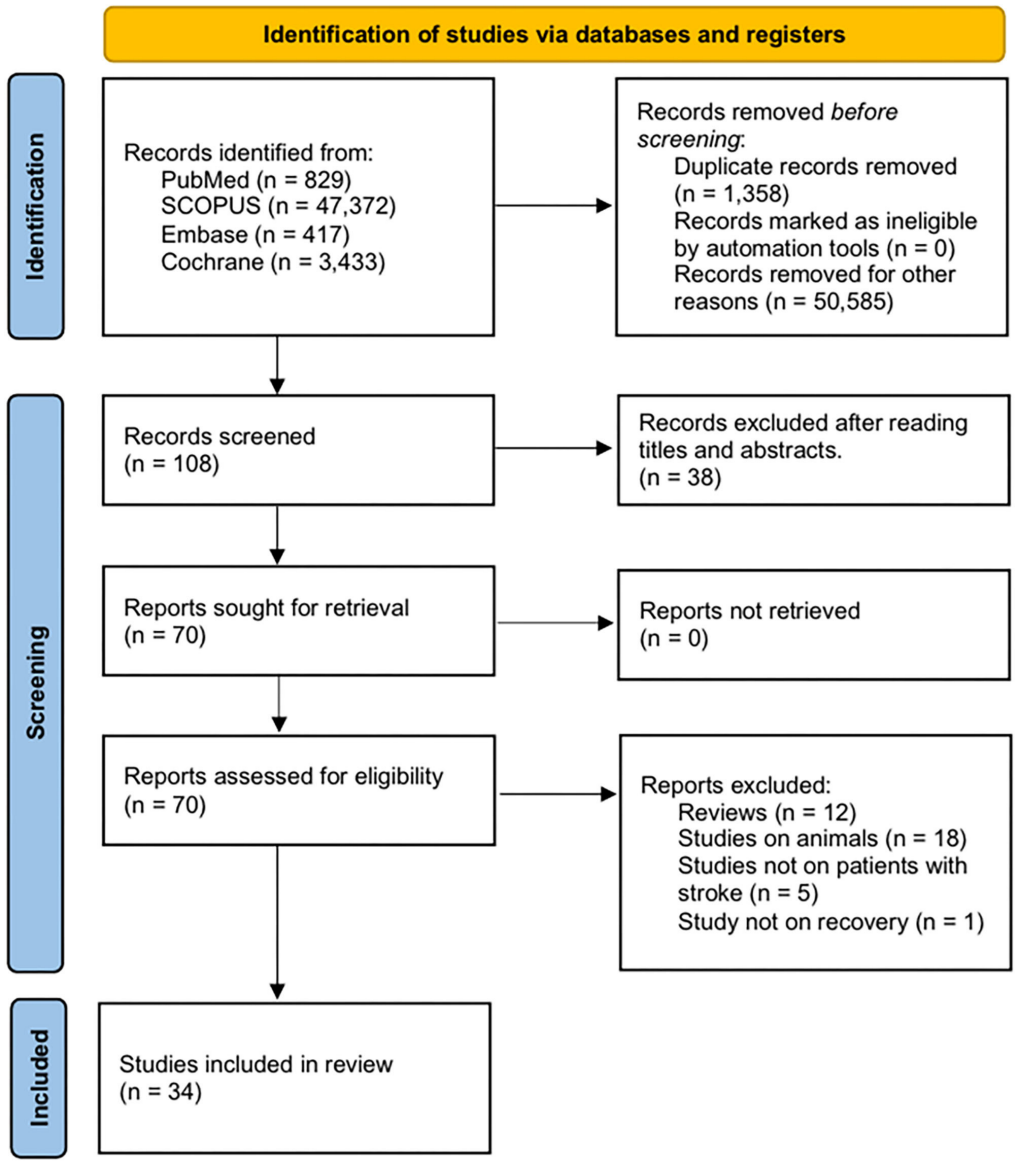
Flow diagram of the study selection process.

The primary literature search yielded a total of 52,051 potentially relevant articles. After reading the titles and abstracts and assessing the eligibility based on complete text, 34 articles were included in this review (Figure 1). Studies were conducted globally, including the United States, Canada, Switzerland, Austria, Australia, the United Kingdom, Italy, Spain, Netherlands, Germany, China, Japan, and South Korea. The characteristics of the included studies are summarized in Table 1. Among the included studies, 14 were on both subtypes of stroke, 13 were on ischemic stroke, four were on hemorrhagic stroke, and three studies did not mention the subtype of stroke included. Of the 34 studies included, only five studies focused on T2DM patients; other studies included both types of diabetes or did not mention specific type of diabetes. The description of diabetes and stroke subtypes, and their diagnostic methods are described in the Supplemental Materials. The results of the quality assessment using the NOS are shown in Table 2, with rates varying from 5 to 8 stars, suggesting moderate to high quality.

**Table 1:** Characteristics of the included studies.

**References**	**Year**	**Type of stroke**	**Type of DM**	**No. of patient**	**Area of recovery**	**Measurement tool**	**Follow up**	**Diabetes or hyperglycemia as the main outcome (Y/N)**	**Main findings**
Megherbi et al. (13)	2003	Ischemic or hemorrhagic	Unspecified	4,481 (DM 937)	Function	Rankin scale, BI	3 m	Yes	The scores of Rankin Scale and BI were significantly higher in diabetics compared to nondiabetics.
Paithankar et al. (18)	2003	Ischemic	Unspecified	72	Function	mRS	3 m	Yes	The presence of diabetes was not associated with bad functional recovery (mRS 4–6) in 72 patients with AIS.
Karapanayiotides et al. (19)	2004	Ischemic or hemorrhagic	Type 1 and type 2	4,064 (DM 611)	Function	Five grade ADL scale	1 m	Yes	Diabetes had no association with poor functional outcome in stroke patients.
Ng et al. (14)	2005	Ischemic or hemorrhagic	Unspecified	92	Function	FIM	Discharge	Yes	Higher total FIM scores at discharge were associated with the absence of DM at discharge, whereas lower FIM scores at discharge were associated with the presence of DM.
Stollberger et al. (15)	2005	Ischemic or hemorrhagic	Unspecified	992 (DM 296)	Function	BI, RS	Discharge	Yes	The BI increased from 45 to 75 in diabetic patients whereas it increased from 50 to 90 in non-diabetics. The proportion of patients in RS score 0–1 was lower in diabetics compared to non-diabetics.
Hankey et al. (27)	2007	Ischemic or TIA	Unspecified	1,662	Function	mRS	18 m	No	Patients without the history of diabetes were more likely to recover from a disable to a non-disabled state after stroke.
Newman et al. (28)	2007	Ischemic	Unspecified	3,118	Cognition and function	MMSE, mRS	1, 2 yrs	No	DM was one of the independent predictors of poorer cognitive function and greater disability.
Ripley et al. (29)	2007	Ischemic or hemorrhagic	Unspecified	367 (DM 114)	Motor and cognition	FIM	1 m	Yes	Diabetes did not significantly impact short-term rehabilitation motor and cognitive outcomes after stroke.
Patel et al. (30)	2007	Ischemic or hemorrhagic	Unspecified	397	QOL	SF-36 (PHSS and MHSS)	1, 3 yrs	No	DM was one of the predictors of poor physical health, measured by SF-36.
Tuttolomondo et al. (20)	2008	Ischemic	Type 2 only	306 (DM 102)	Function	mRS	6 m	Yes	No significant differences were found between diabetics and non-diabetics regarding functional outcome measured with mRS after 6-month follow up.
Dallmeijer et al. (31)	2009	Unspecified	Unspecified	198	Function	mRS	6 m	No	Diabetes had no significant influence on the course of physical functioning, which was measured at 6 months, 1 and 3 years after stroke.
Graham et al. (32)	2009	Ischemic or hemorrhagic	Unspecified	135,097	Function	FIM	Discharge	Yes	Patients under 60 years of age showed that tier diabetes were associated with low FIM discharge scores, but it did not in older stroke patients.
Nannetti et al. (7)	2009	Ischemic or hemorrhagic	Type 2 only	395 (DM 93)	Motor and Function	Fugyl-Meyer, mobility part of MA, BI	Discharge and 1 m	Yes	Diabetes had no overall influence on motor and functional outcome after stroke.
Wei et al. (33)	2010	Ischemic or hemorrhagic	Unspecified	6,354	Function	mRS	12 m	No	Poor functional outcome was strongly associated with diabetes at 12 months in patients with AIS.
Koennecke et al. (34)	2011	Ischemic or hemorrhagic	Unspecified	16,518	Function	mRS	Discharge	No	DM was one of the factors associated with an increased risk of poor outcome.
Jang et al. (35)	2011	Hemorrhagic	Unspecified	281	Function	mRS	3 m	No	A history of diabetes was one of the predictors of functional recovery at 90 days after primary pontine hemorrhage.
Pierni-Yoder et al. (36)	2013	Unspecified	Unspecified	12,155	Function	FIM, length of stay	NA	Yes	Significant differences in functional status was observed in diabetes groups and age showed a significant interaction effect with diabetes status.
Tanaka et al. (37)	2013	Ischemic	Mostly type 2	242 (DM 140)	Function	mRS	1 m	Yes	The risks of poor outcome (mRS 2–6) and END were significantly higher in the diabetics compared to nondiabetics.
Galanth et al. (24)	2014	Ischemic or hemorrhagic	Unspecified	78	QOL	SF-36	1 yr	No	DM was one of the factors which was associated with poor QOL.
Lei et al. (38)	2015	Ischemic	Unspecified	1,877 (DM 526)	Function	mRS	3 m and 1yr	Yes	Elevated HbA1c levels were associated with poor outcome in both diabetics and non-diabetics.
Roquer et al. (39)	2015	Ischemic	Unspecified	1,088 (DM 421)	Function	mRS	3 m	Yes	Diabetes was an independent factor for poor outcome and END.
Ullberg et al. (40)	2015	Ischemic or hemorrhagic	Unspecified	35,064	Function	ADL questionnaire	3, 12 m	No	DM was one of the predictive factors of ADL dependency between 3 and 12 months after stroke.
Wang et al. (21)	2015	Hemorrhagic	Unspecified	1,438 (DM 118)	Function	mRS	1,3,6,12m	Yes	Functional outcome was similar between patients with and without diabetes.
Lattanzi et al. (41)	2016	Ischemic	Type 1 and type 2	112	Function	mRS	3 m	Yes	Increasing HbA1c values were associated with the risk of poor functional outcome at 3 months and the glycemic control (HbA1c ≥ 7%) before stroke occurrence was an independent predictor of unfavorable outcome.
Saxena et al. (16)	2016	Hemorrhagic	Unspecified	2,653 (DM 295)	Function	mRS	3 m	Yes	A history of diabetes was an independent predictor of poor outcome and major disability in patients with ICH.
Tang et al. (17)	2016	Ischemic	Unspecified	419 (DM 98)	Function	mRS	3 m	Yes	Diabetes was an independent factor for unfavorable neurologic outcome (defined by changes in NIHSS and mRS scores) at 24 h, at day 7, and at 3 months.
Kabboord et al. (42)	2018	Unspecified	Unspecified	175	Function	BI	NA	Yes	Diabetes and functional status were independent contributing factors of developing intercurrent diseases druing stroke rehabilitation.
Li and Li (25)	2018	Ischemic	Type 1 and type 2	216	Function	mRS	6 m	Yes	Poor outcome was significantly associated with diabetic microvascular complications.
Mapoure et al. (43)	2018	Ischemic or hemorrhagic	Mostly type 2	701	Function	mRS	3 m	Yes	Patients who were newly diagnosed with diabetes were more likely to have a significantly higher poor functional outcome scores than patients who were previously diagnosed with diabetes.
Ahktar et al. (44).	2019	Ischemic	Type 1 and type 2	2,961 (DM 1,695)	Function	mRS	Discharge and 3 m	Yes	Poor functional outcome was significantly higher in diabetic patients compared with non-diabetics.
Moon et al. (45)	2019	Ischemic	Type 2 only	100 (DM 32)	Motor	MI, MBC, FAC	6 m	Yes	In patients who had interrupted corticospinal tract, motor function recovery was impaired in patients with diabetes compared to those without diabetes.
Wang et al. (46)	2019	Ischemic	Unspecified	408	Function	mRS	3 m	Yes	The patients with poor outcome had higher HbA1c level and diabetes rates than patients with favorable outcome.
Chaturvedi et al. (47)	2020	Ischemic or hemorrhagic	Type 2 only	204 (DM 104)	Function and QOL	FIM, QOL scale	6 m	Yes	Significant improvement in function and QOL were observed in non-diabetics compared to diabetics.
Jang et al. (48)	2020	Hemorrhagic	Type 2 only	221	Motor	MI, MBC, FAC	6 m	Yes	The presence of diabetes did not significantly affect motor outcomes.

*ADL, activity of daily living; BI, Bartheal Index; DM, diabetes mellitus; END, early neurological deterioration; FAC, functional ambulation category; FIM, Functional Independence; ICH, intracerebral hemorrhage; QOL, quality of life; Measure, MBI, modified Barthel Index; MA, motor assessment; MBC, modified Brunnstrom classification; MHSS, mental health summary scores; MI, moticity index; MMSE, Mini-Mental State Examination; NIHSS, National Institutes of Health Stroke Scale; PHSS, physical health summary scores; SF-36, Short Form-36; TIA, transient ischemic attack*.

**Table 2:** Risk of bias assessment by Newcastle-Ottawa Quality Assessment Scale for cohort studies.

**No**.	**References**	**Year**	**Selection**				**Comparability**	**Outcome**			**Quality score**
			**Representativeness of the exposed cohort**	**Selection of the non exposed cohort**	**Ascertainment of exposure**	**Demonstration that outcome of interest was not present at start of study**	**Comparability of** **cohorts on the basis of the design or analysis**	**Assessment of outcome**	**Was follow-up long enough for outcomes to occur**	**Adequacy of follow up of cohorts**	**(total** **score = 9)**
1	Ahktar (44)	2019	⋆	⋆	⋆	⋆	⋆⋆		⋆		7
2	Chaturvedi (47)	2020		⋆	⋆	⋆	⋆⋆		⋆		6
3	Dallmeijer (31)	2009		⋆	⋆		⋆⋆	⋆	⋆	⋆	7
4	Galanth (24)	2014	⋆		⋆		⋆	⋆	⋆	⋆	6
5	Graham (32)	2009	⋆	⋆	⋆		⋆⋆	⋆		⋆	7
6	Hankey (27)	2007	⋆		⋆		⋆⋆	⋆	⋆		6
7	Jang (35)	2011		⋆	⋆		⋆⋆	⋆	⋆	⋆	7
8	Jang (48)	2020		⋆	⋆	⋆	⋆⋆	⋆	⋆	⋆	8
9	Kabboard (42)	2018	⋆	⋆	⋆		⋆⋆	⋆	⋆	⋆	8
10	Karapanayiotides (19)	2004	⋆	⋆	⋆		⋆⋆	⋆			6
11	Koennecke (34)	2011	⋆		⋆		⋆⋆	⋆	⋆	⋆	7
12	Lattanzi (41)	2016		⋆	⋆		⋆⋆	⋆	⋆	⋆	7
13	Lei (38)	2015	⋆	⋆	⋆		⋆⋆	⋆	⋆	⋆	8
14	Li and Li (25)	2018		⋆	⋆		⋆⋆	⋆	⋆	⋆	7
15	Mapoure (43)	2018		⋆	⋆		⋆⋆	⋆	⋆	⋆	7
16	Megherbi (13)	2003	⋆	⋆	⋆		⋆⋆	⋆	⋆	⋆	8
17	Moon (45)	2019		⋆	⋆	⋆	⋆⋆	⋆	⋆	⋆	8
18	Nannetti (7)	2009	⋆	⋆	⋆	⋆	⋆	⋆		⋆	7
19	Newman (28)	2007	⋆		⋆		⋆⋆	⋆	⋆	⋆	7
20	Ng (14)	2005			⋆		⋆⋆	⋆		⋆	5
21	Paithankar (18)	2003			⋆		⋆	⋆	⋆	⋆	5
22	Patel (30)	2007	⋆		⋆		⋆⋆	⋆	⋆	⋆	7
23	Piernik-yoder (36)	2013	⋆	⋆	⋆		⋆⋆	⋆		⋆	7
24	Ripley (29)	2007		⋆	⋆		⋆⋆	⋆		⋆	6
25	Roquer (39)	2014	⋆	⋆	⋆		⋆⋆	⋆	⋆	⋆	8
26	Saxena (16)	2016	⋆	⋆	⋆		⋆⋆	⋆	⋆	⋆	8
27	Stollberger (15)	2005	⋆	⋆	⋆		⋆⋆	⋆		⋆	7
28	Tanaka (37)	2013		⋆	⋆	⋆	⋆⋆	⋆		⋆	7
29	Tang (17)	2015		⋆	⋆		⋆⋆	⋆	⋆	⋆	7
30	Tuttolomondo (20)	2008			⋆		⋆⋆	⋆	⋆	⋆	6
31	Ullberg (40)	2014	⋆	⋆	⋆		⋆⋆		⋆	⋆	7
32	Wang (21)	2015		⋆	⋆		⋆⋆		⋆	⋆	6
33	Wang (46)	2019		⋆	⋆		⋆⋆	⋆	⋆	⋆	7
34	Wei (33)	2010	⋆		⋆		⋆⋆		⋆	⋆	6

*In the Newcastle-Ottawa scale (NOS), stars awarded for each quality item. Stars are awarded accordingly such that the highest quality studies are awarded up to nine stars*.

### Effects of Diabetes on Recovery of ADL

Studies on the effect of diabetes on the recovery of ADL began in the early 2000s. It was first reported by Megherbi et al. in 2003 (13), wherein patients with diabetes were compared with those without diabetes (937 vs. 3,544), using the mRS and MBI at 3 months after stroke. The results showed that the mRS and MBI scores were significantly higher in patients with diabetes than in those without diabetes. In contrast, in the same year, Paithankar et al.'s study reported that diabetes was not associated with poor ADL recovery (mRS: 4–6) after ischemic stroke (18). A year later, Karapanayiotides et al. also reported that diabetes was not associated with poor ADL recovery in stroke patients (19).

Subsequently, several research groups tried to determine whether diabetes affects ADL recovery after stroke, and many studies have reported the negative impact of diabetes on ADL recovery. In 2005, Ng et al. showed that diabetes was associated with a low FIM score (14). A 2005 study by Stollberger et al. (15) also reported that stroke patients with diabetes showed a poorer ADL recovery than patients without diabetes. The proportion of good recovery was lower in people with diabetes compared to people without diabetes. In 2007, Hankey et al. showed that the absence of diabetes was a significant prognostic factor for good ADL recovery (defined as mRS <3) (27) and Newman et al. reported that diabetes was an independent predictor of greater disability (28). In contrast, other studies have reported that there is no correlation between the presence of diabetes and ADL recovery. In 2008, Tuttolomondo et al. compared 102 stroke patients with diabetes to 204 stroke patients without diabetes and concluded that no significant differences in ADL recovery were found between them for a period of 6 months after the ischemic stroke (20). Similarly, other studies reported that diabetes had no significant influence on the course of ADL after stroke (7, 31). A 2009 study by Graham et al. reported that the effect of age on recovery was more significant than diabetes alone. Their results showed that there was an association between diabetes and FIM scores in patients under 60 years of age but not in those above 60 (32); a similar result was reported in 2014 by Piernik-Yoder et al. (36). In the 2000s, although 6 out of 11 studies (13–15, 27, 28, 32) reported that there were some effects of diabetes on ADL recovery after stroke, these studies in the 2000s yielded contradictory results.

In the 2010s, further studies investigated various prognostic indicators of post-stroke recovery and revealed that diabetes was one of the important predictors associated with ADL recovery. In 2010, Wei et al. compared recovery patterns and changes in ADL in 6,354 patients with ischemic stroke or intracerebral hemorrhage (ICH) (33) and reported that a poor ADL (mRS ≥ 3) at 12 months after ischemic stroke was strongly associated with diabetes. Similarly, other studies reported that a history of diabetes was one of the predictive factors of ADL recovery at 3 months (35, 40), as well as one of the factors associated with poor ADL recovery at discharge (34). In 2013, Tanaka et al. reported that the risk of poor recovery after stroke was higher in 104 patients with diabetes compared to 102 patients without diabetes (37), in contrast to the study by Lei et al., which reported that poor recovery was associated with elevated levels of HbA1c, regardless of the presence or absence of diabetes (38). Studies by Roquer et al. (39), Saxena et al. (16), and Tang et al. (17) also reported that diabetes was an independent predictor of poor outcome and major disability at 3 months after stroke. In 2016, Lattanzi et al. suggested that increased HbA1c values were associated with the risk of poor ADL at 3 months after stroke in patients with diabetes (41). High glycemic control (HbA1c ≥ 7%) before stroke occurrence was an independent predictor of unfavorable outcomes; better glycemic control before stroke onset is recommended to improve the prognosis of stroke patients with diabetes.

Additionally, some studies have focused on the complications caused by diabetes and highlighted the importance of the timing of diabetes diagnosis. A 2018 study by Li et al. showed that poor recovery was significantly associated with diabetes-specific microvascular complications (25), and Kabboard et al. showed that a low functional status (defined as Barthel index ≤ 14) on admission and the presence of comorbidities, particularly diabetes, were independent contributing factors for developing intercurrent diseases, such as cardiovascular or psychiatric diseases (42). Mapoure et al. showed that patients who were newly diagnosed with diabetes were more likely to have a significantly poorer ADL recovery at 3 months after stroke (mRS > 2) than patients who were previously diagnosed with diabetes, thereby suggesting that the timing of diabetes diagnosis is also important (43). Further studies have continuously demonstrated the effect of diabetes on recovery of ADL by adjusting for critical factors, such as age, stroke subtype, and other comorbidities, such as hypertension and atrial fibrillation. In 2019, Akhtar et al. reported that the percentage of diabetic patients with poor ADL (mRS: 3–6) after ischemic stroke, at discharge and at 3 months, was significantly higher than that of prediabetic and non-diabetic patients (44). In the same year, a study by Wang et al., involving 408 acute ischemic stroke patients, also reported that patients with poor ADL (mRS: 2–6) at 3 months after ischemic stroke showed higher HbA1c levels and diabetes rates than patients with better ADL (46). This study showed that patients with HbA1c > 5.7% were more susceptible to poorer ADL (mRS: 2–6) at 3 months after ischemic stroke than patients with HbA1c <5.7%. Recently, Chaturvedi et al. directly compared ADL recovery in stroke patients with type 2 diabetes and those without type 2 diabetes (104 patients in each group) (47). Significant improvement in ADL was seen after 6 months in patients without diabetes when compared to patients with diabetes, represented by higher FIM scores. In the diabetic group, the relative risk (RR) of poor ADL recovery was 1.34, with an odds ratio (OR) of 1.8. In summary, 16 out of 18 studies in the 2010s (16, 17, 25, 33–37, 39–44, 46, 47) reported that poor ADL recovery was associated with diabetes and that diabetes was one of the predictors of poor ADL recovery after stroke. Overall, 22 out of 29 studies supported that diabetes was associated with impaired ADL recovery after stroke and suggested the possibility of the negative influence of diabetes on ADL recovery in stroke patients.

### Effect of Diabetes on Motor Recovery

Our search yielded four studies on the effect of diabetes on motor recovery. The first study on the impact of diabetes on post-stroke motor recovery was conducted in 2007 by Ripley et al., which reported that diabetes was not a significant predictor of acute rehabilitation motor outcomes (29). In 2009, Nannetti et al. investigated the effect of diabetes on motor recovery in 395 stroke patients (93 patients with diabetes vs. 302 patients without diabetes) (7). Mobility and motor function were assessed using the mobility part of the motor assessment chart according to Lindmark and Hamrin and the FMA scale, respectively. Patients in both groups showed a progressive improvement in all outcome measures, and diabetes had no influence on motor recovery after stroke. In 2019, Moon et al. conducted a study using diffusion tensor tractography to assess the integrity of the corticospinal tract (CST), which is the most important structure for motor function (45). This study only recruited patients with corona radiate infarction, adjusting the infarct location (100 stroke patients; 32 with diabetes vs. 68 without diabetes), and classified patients according to the integrity of the CST, which is a critical factor that could affect motor recovery. Motor recovery was assessed at 6 months after stroke using upper and lower limb MI, MBC, and FAC. The results showed that among the patients with interrupted CST, motor recovery was impaired in those with diabetes compared to those without diabetes. Interestingly, when CTS was preserved, motor outcomes were favorable in both patients with or without diabetes. However, in this study, the authors did not consider the influence of lesion size, which is another important factor that can affect motor outcomes. In 2020, a retrospective study by Jang et al., which had a study design similar to that of Moon et al., but with adjusted confounding factors (including the state of the CST, age, lesional volume, and treatment method), reported contrasting results (48). In this study, the results of motor outcomes, which were measured using the upper and lower limb MI, MBC, and FAC at 6 months in 221 patients with basal ganglia ICH, showed that the presence of diabetes did not significantly affect motor outcomes.

Among the four studies on motor recovery, only one (45) showed that diabetes had some influence on motor recovery (7, 29, 48). Although the results of the included studies suggest that diabetes does not seem to hinder motor recovery prominently, the effect of diabetes on motor recovery after stroke remains controversial due to the small number of studies.

### Effect of Diabetes on Cognitive Recovery

Our search yielded two studies on the impact of diabetes on cognitive recovery (28, 29). In 2007, Ripley et al. reported that diabetes did not significantly affect short-term rehabilitation cognitive outcomes after stroke, which were measured using the FIM cognitive score (29). In contrast, Newman et al. reported that diabetes was associated with cognitive recovery, which was assessed using the MMSE (28). Diabetes was one of the independent predictors of poor cognitive recovery, lower high-density lipoprotein, and higher homocysteine levels, suggesting that these metabolic disturbances are risk factors for progressive vascular impairment that could influence cognitive recovery. Since we found only two studies on cognitive recovery, it is inconclusive whether diabetes has an impact on cognitive recovery. Further well-controlled prospective studies are needed to clarify the effect of diabetes on cognitive recovery.

### Effect of Diabetes on QOL

In 2007, Patel et al. investigated the predictive factors associated with health-related QOL using the physical health summary scale of the SF-36 (30). This study, involving 397 stroke patients, revealed that diabetes was a predictor of poor physical health. In 2014, Galanth et al. also investigated factors that affected the QOL of 78 stroke patients 1 year after stroke, using the SF-36 QOL questionnaires (24). Changes were observed in all aspects of life, and diabetes was one of the factors associated with poor QOL. Furthermore, in 2020, Chaturvedi et al. reported that significant improvement was observed in the QOL after 6 months (measured with stroke-specific QOL scores) in patients without diabetes compared to those with diabetes. In the diabetic group, the RR of poor QOL was 1.56, with an OR of 2.83. Although only a few studies have been conducted on QOL, diabetes seems to have some effect on QOL in stroke patients.

## Discussion

**Figure 2:**
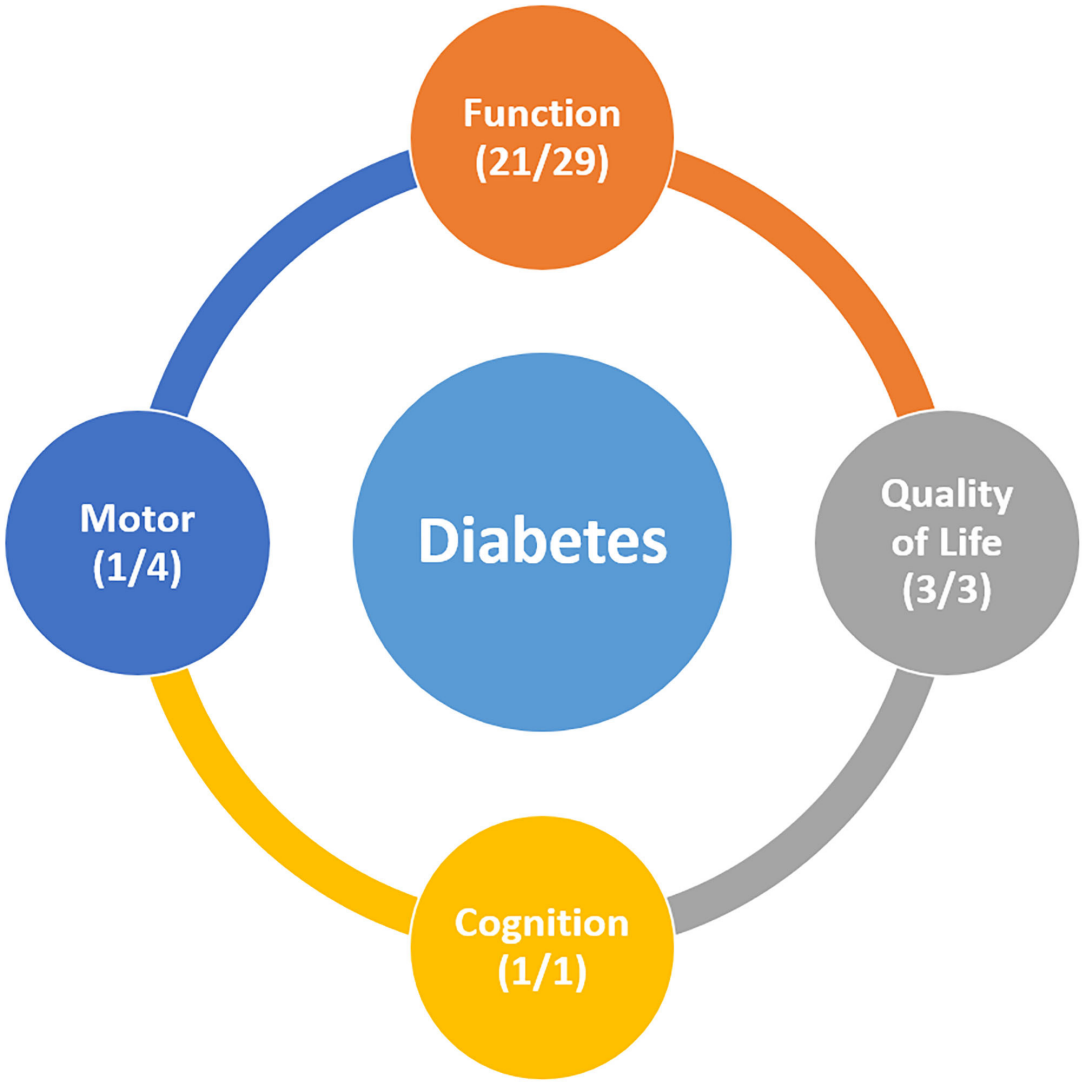
(A) studies which reported that diabetes has a significantly negative effect on post-stroke recovery (B) all studies reporting the relationship between diabetes and post-stroke recovery.

In this review, we aimed to investigate the effect of diabetes on recovery after stroke. Overall, the results of our review suggests that diabetes has some impact on post-stroke recovery. Out of the 29 studies on recovery of ADL, 22 suggested that diabetes had a negative effect on recovery of ADL after stroke. Many studies have shown that diabetes is associated with impaired ADL recovery after stroke, even after adjusting for factors such as age, stroke subtype, and other comorbidities. Importantly, studies including large sample size showed that diabetes was one of the predictive factors of ADL dependency after stroke (13, 40). Regarding motor recovery, only one out of four studies showed that diabetes had some effect on motor recovery after stroke. Of the two studies on cognitive recovery, one reported that diabetes was an independent predictor of poor cognitive recovery after stroke. Three studies on QOL reported that a poor QOL after stroke was associated with the presence of diabetes. Although the evidence is insufficient to draw a conclusion due to the small number of studies, diabetes seems to have some influence on QOL but not prominently on motor or cognitive recovery (Figure 2). Overall, our review highlights the potential role of diabetes, which may lead to poorer clinical outcomes after stroke. Of the 34 included studies, 27 demonstrated that diabetes was somewhat negatively associated with recovery after stroke. Although the exact mechanism underlying this phenomenon remains unclear, there appears to be a relationship between increasing HbA1c levels and poorer recovery after stroke.

Stroke is the leading cause of disability, often limiting functional abilities, daily activities, and mobility (49). Inflammatory conditions, such as obesity and diabetes contribute to atherosclerosis, cardiovascular disease, peripheral nerve dysfunction, sarcopenia, muscle fat infiltration, and decreased physical activity (50–52). Thus, presence of comorbidities, such as obesity and diabetes, increase the risk for functional disability in stroke patients and greater amount of disability can occur in stroke patients in the presence of these conditions (53). After stroke, an adequate supply of glucose to the brain is important for maintaining brain function (54). Cerebral glucose metabolism is required for brain energy maintenance and neurotransmitter synthesis. Substances like acetylcholine, glutamate, glycine, and γ-amino butyric acid are synthesized through glucose metabolic pathways, indicating that neurotransmission and energy metabolism are closely interrelated (47). Diabetes is characterized by defects in insulin secretion and action, leading to inefficient glucose transport and metabolism in target organs (54). In diabetes, a disruption of systemic glucose metabolism and impairment of glucose supply to the brain occur, which may cause abnormalities in brain metabolism and function.

There may be several possible explanations for the effect of diabetes on ADL recovery. One possible explanation may be the impairment of neurogenesis and neuroplasticity in patients with diabetes after stroke. Several preclinical studies have suggested an association between diabetes and impaired neurogenesis and neuroplasticity. Previous animal models suggested that a time-limited window of neuroplasticity opens following a stroke via neuroplasticity mechanisms which include synapse strengthening and activity-dependent rewiring (55). During this period, the greatest gains in recovery occur through neuroplasticity. Neuroplasticity is achieved by increased neurogenesis and generation of new neurons from progenitors, which contributes to the reshaping of the damaged brain (56). Reduced neuroplasticity after stroke has been reported in several animal models of diabetes (4, 57, 58). Impaired neuroplasticity correlated with reduced neurogenesis, persistent atrophy of GABAergic parvalbumin–positive interneurons, which are important contributors to neuroplasticity after injury (4, 59). In addition, the somatostatin-expressing neurons, another contributor of neuroplasticity, was also affected by diabetes during the stroke recovery phase (58). Another animal study reported that after stroke, when compared to non-diabetic mice, hyperglycemic mice showed greater impairment of sensory function, less cortical responses to touch, and a greater decrease in axonal density, leading to impaired neuroplasticity (9, 60). In the hyperglycemic mice, persistent behavioral deficits in sensorimotor function and absence of functional reorganization of the cortex was noted (9). A recent study involving stroke patients with diabetes showed the absence of ipsilesional cortical excitability change after diabetic stroke, implying impaired neuroplasticity over the ipsilesional hemisphere (61).

In addition, impaired angiogenesis has been reported in patients with diabetes after stroke (62). Pulsinelli et al. explained that the neurological outcome was poorer in patients with diabetes compared to patients without diabetes, possibly due to the presence of proliferative angiopathy of small cerebral blood vessels or severe cerebral arteriosclerosis, which could interfere with collateral blood flow to the peri-ischemic zone of the cerebral infarct (63). During stroke, compensatory neovascularization occurs in the ischemic area, 3–4 days following ischemic insults, to meet the metabolic demand (64). Reparative angiogenesis is important for good functional recovery after stroke (65, 66) and is associated with an increase in cerebral blood volume and cerebral blood flow (67). Chronic glucagon-like peptide-1 receptor activation can stimulate angiogenesis and mediate post-stroke functional recovery by improving vascular remodeling in the recovery phase (68). Diabetic animals showed impaired neovascularization and prominent vascular injury after cerebral ischemia (62, 69). This significant vasoregression leads to the swelling of astrocytes and poor functional recovery (62). As diabetes may have a negative effect on neuroplasticity and angiogenesis, patients with diabetes may be susceptible to poor ADL recovery after stroke (67).

Another possible explanation for the potential harmful effects of diabetes on post-stroke recovery may be associated with brain insulin resistance (70). Insulin plays an important role in the formation of neural circuits and synaptic connections, and it facilitates and promotes neuroplasticity (71). Activation of the insulin receptor and insulin-like growth factor receptor signaling pathways activates the neuronal antioxidant defense mechanism and engages synaptic plasticity mechanisms, thereby promoting recovery after brain injury (72). The brain insulin resistance observed in diabetes is associated with alterations in neural metabolic functions and restorative processes, and it also allows susceptibility to neurodegeneration (70). Poor insulin signaling in neurons may contribute to decreased synaptogenesis and axonal sprouting after stroke, leading to poor ADL recovery (73).

The detrimental effects of hyperglycemia in patients with diabetes may cause further cerebral damage after stroke, which may also contribute to poor ADL recovery. After focal cerebral ischemia, glucose is anaerobically metabolized to lactic acid (74), and the production of lactate increases, leading to irreversible neuronal injury and consequent expansion of the infarct core into the penumbra. Metabolic abnormalities in diabetes can aggravate this process, as hyperglycemia causes an elevation of lactate and H^+^ production, facilitating further cerebral damage. Hyperglycemia triggers free radical production, endonuclease activation, glutamate release, and alteration of intracellular Ca^+^ regulation (75). It can also decrease the activity of tissue plasminogen activator, leading to impaired recanalization, delayed reperfusion, and increased infarct size (76, 77). During the healing process after stroke, hyperglycemia itself can be directly neurotoxic; it can cause reperfusion injury, oxidative stress, alteration of the blood–brain barrier, endothelial dysfunction, and inflammatory responses (28, 78), all of which can trigger further neuronal death. Chronic systemic hyperglycemia in diabetes causes impaired glucose transport and cell-to-cell metabolic interactions, along with changes in the activities of key enzymes involved in glycogen metabolism (54). Hyperglycemia leads to alterations in brain energy and neurotransmitter homeostasis, consequently causing brain injury and dysfunction (54). Peripheral insulin resistance triggers insulin resistance in the brain, leading to hyperglycemia and development of diabetes-related comorbidities (79). As metabolic disturbances in diabetes lead to progressive vascular dysfunction, ischemic damage after stroke may be amplified. Thus, stroke patients with diabetes can be vulnerable to progressive brain damage beyond the initial attack of stroke and are at an increased risk of poor ADL recovery.

Suggestively, motor recovery mechanisms after stroke, including perilesional reorganization and contributions from the secondary motor area (48), may not work properly in patients with diabetes, resulting in poor motor recovery. However, after adjusting for critical factors, such as the state of CST, age, lesional volume, and treatment method, diabetes did not seem to affect motor recovery after stroke (48). It has been suggested that the preservation of the CST or lesional volume is more important for predicting motor prognosis than the presence or absence of diabetes (45). However, due to the small number of studies on the influence of diabetes on motor recovery, it is inconclusive whether diabetes has an influence on motor recovery. Further studies addressing the integrity of the CST and influence of diabetes on motor recovery are needed to validate the effect of diabetes on motor recovery after stroke.

Diabetes causes vascular dysfunction and alterations in neuroplasticity, including impairment of hippocampal neurogenesis, which may contribute to poor cognitive recovery (80). Chronically elevated blood glucose level increases the risk of microstructural changes in the white matter tracts, and poor metabolic control accelerates cognitive decline (81). This may result in decreased performance on tasks that require planning and execution, attention, and learning and memorization (82). Only two studies were found from our research on cognitive recovery, which were insufficient to determine whether diabetes had an impact on cognitive recovery.

Diabetes seems to have some influence on QOL after stroke. QOL refers to a person's individual perception of physical, emotional, and social status (83, 84). Since a complete cure may not be achieved in patients with diabetes, many patients with diabetes reported a lower QOL than healthy individuals (84). Slow recovery associated with the presence of diabetes may aggravate the perception of health status in stroke patients and can negatively affect QOL. Poor recovery can also increase the burden on patients and caregivers, which may result in irritable mood and depression, thereby affecting their QOL (47).

The current review investigated the influence of diabetes on post-stroke recovery. However, this review has some limitations. Although it is possible that many confounding factors, such as age, sex, lesional volume, and body mass index, could have affected the relationship between diabetes and clinical outcomes, the studies included in this review did not consider all these possible confounding factors. Because of the retrospective nature of some studies, it was impossible to investigate the impact of confounding factors. The differences between the studies may be attributable to these possible confounding factors. In addition, only 12 out of 24 studies (13, 19, 21, 24, 25, 37–39, 43, 45, 46, 48) specified how the diagnosis of diabetes was made, including the fasting serum glucose level and HbA1c level; the other studies either did not report how they defined diabetes, or defined diabetes based on the “history” of diabetes. The duration of diabetes was also not mentioned in any of the included studies. Importantly, most studies included in this review did not assess patients according to the type of diabetes separately. Only 5 studies (7, 20, 45, 47, 48) specifically mentioned that they included patients only with T2DM. In addition, nine out of 34 studies (24, 27, 28, 30, 31, 33–35, 40) did not focus on the effect of diabetes on post-stroke recovery, but merely mentioned that diabetes was a risk factor associated with poor post-stroke recovery. Studies that focused on the effect of diabetes on recovery also showed contradictory results. However, overall, it appears that diabetes may negatively influence recovery after stroke. As the burden of both diabetes and stroke increases in the global healthcare system, appropriate methods and timing of screening for diabetes and stroke are necessary to lessen their progressive burden. Whether strict management of diabetes has a positive effect on recovery after stroke could not be assessed in this review, because studies did not investigate the beneficial effects of glycemic control on post-stroke recovery. Further studies on this subject are warranted.

## Conclusion

To summarize, the post-stroke recovery of ADL in patients with diabetes seems to be poorer than that in patients without diabetes. In addition, there are insufficient data to conclude the effect of diabetes on motor and cognitive recovery; however, diabetes seems to have some influence on the QOL after stroke. Impaired neurogenesis, neuroplasticity, and angiogenesis in diabetes and the detrimental effects of hyperglycemia may be associated with poor post-stroke recovery. To elucidate the pathophysiological mechanism of diabetes in post-stroke recovery, well-controlled prospective studies are needed.

## Data Availability Statement

The original contributions presented in the study are included in the article/Supplementary Material, further inquiries can be directed to the corresponding author.

## Author Contributions

SY, MB-R, SK, ML, and MC: conceptualization, methodology, writing-original draft, writing-review, and editing. MC: supervision. All authors contributed to the article and approved the submitted version.

## Funding

The present study was supported by a National Research Foundation of Korea grant funded by the Korean Government (grant no. NRF-2019M3E5D1A02069399).

## Conflict of Interest

The authors declare that the research was conducted in the absence of any commercial or financial relationships that could be construed as a potential conflict of interest.

## Publisher's Note

All claims expressed in this article are solely those of the authors and do not necessarily represent those of their affiliated organizations, or those of the publisher, the editors and the reviewers. Any product that may be evaluated in this article, or claim that may be made by its manufacturer, is not guaranteed or endorsed by the publisher.
