# Supplementary material for: Effect of Diabetes on Post-stroke Recovery: A Systematic Narrative Review
Source: Front Neurol. 2021 Dec 14;12:747878. doi: 10.3389/fneur.2021.747878 (PMC8712454; doi:10.3389/fneur.2021.747878)
Supplement: Supplementary file 1 [file Data_Sheet_1.docx]

**Supplemental Materials**

Supplemental Table 1. Characteristics of the included studies

| **First author** | **Year** | **Country** | **Diagnosis of stroke** | **Diagnosis of DM** |
| --- | --- | --- | --- | --- |
| Megherbi et al. | 2003 | England, France, Germany, Hungry, Italy, Portugal, and Spain | Stroke was defined according to the World Health Organization (WHO) with brain imaging confirmation. | Patients with repeated fasting plasma  glucose level performed in all centers > 7.8 mmol/L (140 mg/dL) were enrolled in accordance with the WHO diagnostic criteria for diabetes used in 1993. |
| Paithankar et al. | 2003 | India | CT scan | Unspecified |
| Karapanayiotides et al. | 2004 | Switzerland | Infarct topography was specified by CT or MRI. | Patients were defined as diabetic if they had known DM before stroke according to the new diagnostic criteria of the Expert Committee on the Diagnosis and Classification of Diabetes Mellitus (fasting glucose level of ≥7.0 mmol/L or a plasma glucose level of ≥11.1 mmol/L after glucose ingestion. |
| Ng et al. | 2005 | United States | Stroke was deﬁned based on clinical features consistent with stroke and supported by CT or MRI ﬁndings. | Unspecified |
| Stollberger et al. | 2005 | Austria | The Austrian Stroke Registry (All consecutive patients with acute stroke were documented and evaluated.) | Diabetes was assessed as present in patients with a history of diabetes or treatment with antidiabetic drugs. |
| Hankey et al. | 2007 | Australia | Not specified | Unspecified |
| Newman et al. | 2007 | United States | CT or MRI studies | Unspecified |
| Ripley et al. | 2007 | United States | Only patients with clear radiographic evidence of single-hemisphere lesions were included. Stroke diagnosis was based on coding of rehabilitation admission diagnosis. | Diabetes diagnosis was based on coding of past med-  ical history comorbidities from admission history  documentation. |
| Patel et al. | 2007 | United Kingdom | Stroke was deﬁned according to the WHO criteria | Unspecified |
| Tuttolomondo et al. | 2008 | Italy | Stroke was deﬁned as a clinical  syndrome of rapidly developing symptoms or signs of focal loss of cerebral function with symptoms which had lasted more than 24 h and had no apparent cause other than vascular origin. | Patients were deﬁned as type 2 diabetics if they  had known diabetes treated by diet, oral hypoglycemic drugs or insulin before stroke. |
| Dallmeijer et al. | 2009 | Netherlands | Unspecified | Unspecified |
| Graham et al. | 2009 | United States | ICD-9 codes | ICD-9 codes |
| Nannetti et al. | 2009 | Italy | Diagnosis made by a neurologist; definition includes  a sudden, nonconvulsive, focal neurological deficit persisting for more than 24 h. | According to the American Diabetes Association criteria |
| Wei et al. | 2010 | China | Unspecified | Unspecified |
| Koennecke et al. | 2011 | Germany | Unspecified (Directed by neurologists) | Pathologic elevated fasting blood glucose level or history of diabetes |
| Jang et al. | 2011 | South Korea | CT imaing | History of diabetes |
| Pierni-Yoder et al. | 2013 | United States | ICD-9 codes | ICD-9 codes |
| Tanaka et al. | 2013 | Japan | CT or MRI studies | A 75-g oral glucose tolerance test (141 diabetes, 52 patients prediabetes, 50 normal patients) |
| Galanth et al. | 2014 | French West Indies | CT or MRI studies | Fasting capillary glycemia up to 1.26g/L, HbA1c up to 6.5%, or antidiabetic treatment |
| Lei et al. | 2015 | China | Unspecified | The use of antidiabetic medication, a fasting serum glucose level ≥7.0 mmol/L, a non-fasting glucose concentration ≥11.1 mmol/L, or a self-reported physician diagnosis |
| Roquer et al. | 2015 | Spain | CT or MRI studies, additional evaluations if needed | non-DM (HbA1c during admission<5.7 % and no previous evidence of 2 or more fasting glucose>126 mg/dL), prediabetes (HbA1c from 5.7 to 6.4 %), and DM (previous DM diagnosis or HbA1c≥6.5 % independently of current blood glucose) |
| Ullberg et al. | 2015 | Sweden | ICD-10 codes (Swedish Stroke Register) | Unspecified |
| Wang et al. | 2015 | China | CT or MRI studies; Clinical diagnosis of stroke according to  The deﬁnition recommended by the American Heart Association and American  Stroke Association | Diabetes was defined as fasting plasma glucose ≥126mg/dl on repeated measurement; postload plasma glucose >198mg/dl; patient self-report of  diabetes; or history of anti-diabetic therapy |
| Lattanzi et al. | 2016 | Italy | Unspecified | Diabetes was deﬁned according to patients’ self-report history or the use of hypoglycemic drug or insulin |
| Saxena et al. | 2016 | Australia | CT scan | A history of diabetes and level of blood glucose |
| Tang et al. | 2016 | China | CT or MRI studies | Diabetes was deﬁned as a history of diabetes and new diagnosis according to the standard of WHO 2011. |
| Kabboord et al. | 2018 | Netherlands | Unspecified | Unspecified |
| Li et al. | 2018 | China | CT and MRI studies | The diagnosis of diabetes mellitus included both known pre-existing diabetes and newly diagnosed diabetes. Patients were newly diagnosed with diabetes according to the following criteria: fasting plasma glucose level of > 7.0 mmol/l (126 mg/dl), symptoms of hyperglycemia and a casual plasma glucose level of > 11.1 mmol/l (200 mg/dl), and a 2-hour plasma glucose level of > 11.1 mmol/l (200 mg/dl) during a 75-g oral glucose tolerance test. |
| Mapoure et al. | 2018 | Cameroon | A neurological assessment done by a neurologic or intenstive care specialist and CT scans | Diabetes was deﬁned as a random plasma glucose level of 200 mg/dL or higher and/or in-hospital fasting glucose of 126 mg/dL or higher on 2 or more occasions and/or glycated hemoglobin (HbA1c) of 6.5% or higher. |
| Ahktar et al. | 2019 | Canada | Stroke diagnosis conﬁrmed using the ICD-10 deﬁnitions | HbA1c was used according to the 2018 American Diabetes Association recommendations to categorize patients into those without diabetes (<5.7%), prediabetes (5.7%-6.4%), and diabetes (≥6.5%). |
| Moon et al. | 2019 | South korea | An infarct at the level of the corona radiata  explaining motor weakness determined by brain MRI with confirmation by a neuro-radiologist | Fasting plasma glucose level ≥126 mg/dL, plasma glucose at 2 hours after 75-g oral glucose load ≥200 mg/dL, HbA1c ≥6.5%, random plasma glucose ≥200 mg/dL with classic hyperglycemic  symptoms, and previous diagnosis of type 2 iabetes |
| Wang et al. | 2019 | China | Stroke was conﬁrmed by clinical symptoms and neuroimages according to the recommendations from WHO. | Diabetes was diagnosed as use of antidiabetic medicines, previous physician diagnosis, or HbA1c  ≥6.5%. |
| Chaturvedi et al. | 2020 | India | Unspecified | Unspecified |
| Jang et al. | 2020 | South Korea | A hematoma seen on brain CT with conﬁrmation by a neuroradiologist | Fasting plasma glucose level ≥126 mg/dL, plasma glucose at 2 h after 75 g oral glucose load ≥ 200 mg/dL, HbA1c ≥ 6.5%, random plasma glucose ≥ 200 mg/dL with classic hyperglycemic symptoms, and previous diagnosis of type 2 diabetes |

**Abbreviations**

ADL: activity of daily living, BI: Bartheal Index, DM: diabetes mellitus, END: early neurological deterioration, FAC: functional ambulation category, FIM: Functional Independence, HbA1c: hemoglobin A1c, ICH: intracerebral hemorrhage, QOL: quality of life, Measure, MBI: modified Barthel Index, MA: motor assessment, MBC: modiﬁed Brunnstrom classiﬁcation, MHSS: mental health summary scores, MI: moticity index, MMSE: Mini-Mental State Examination, NIHSS: National Institutes of Health Stroke Scale, PHSS: physical health summary scores, SF-36: Short Form-36, TIA: transient ischemic attack
